# Immunoscreening of the extracellular proteome of colorectal cancer cells

**DOI:** 10.1186/1471-2407-10-70

**Published:** 2010-02-25

**Authors:** Susanne Klein-Scory, Salwa Kübler, Hanna Diehl, Christina Eilert-Micus, Anke Reinacher-Schick, Kai Stühler, Bettina Warscheid, Helmut E Meyer, Wolff Schmiegel, Irmgard Schwarte-Waldhoff

**Affiliations:** 1 Department of Internal Medicine, Knappschaftskrankenhaus, IMBL, Ruhr-University Bochum, Bochum, Germany; 2 Medical Proteome-Center, Ruhr-University Bochum, Bochum, Germany; 3 Clinical & Cellular Proteomics, Medical Faculty/Centre for Medical Biotechnology, Duisburg-Essen University, Essen, Germany; 4 Department of Internal Medicine, Knappschaftskrankenhaus, Ruhr-University Bochum, Bochum, Germany; 5 Department of Gastroenterology and Hepatology, Kliniken Bergmannsheil, Ruhr-University Bochum, Bochum, Germany

## Abstract

**Background:**

The release of proteins from tumors can trigger an immune response in cancer patients involving T lymphocytes and B lymphocytes, which results in the generation of antibodies to tumor-derived proteins. Many studies aim to use humoral immune responses, namely autoantibody profiles, directly, as clinical biomarkers. Alternatively, the antibody immune response as an amplification system for tumor associated alterations may be used to indicate putative protein biomarkers with high sensitivity. Aiming at the latter approach we here have implemented an autoantibody profiling strategy which particularly focuses on proteins released by tumor cells in vitro: the so-called secretome.

**Methods:**

For immunoscreening, the extracellular proteome of five colorectal cancer cell lines was resolved on 2D gels, immobilized on PVDF membranes and used for serological screening with individual sera from 21 colorectal cancer patients and 24 healthy controls. All of the signals from each blot were assigned to a master map, and autoantigen candidates were defined based of the pattern of immunoreactivities. The corresponding proteins were isolated from preparative gels, identified by MALDI-MS and/or by nano-HPLC/ESI-MS/MS and exemplarily confirmed by duplex Western blotting combining the human serum samples with antibodies directed against the protein(s) of interest.

**Results:**

From 281 secretome proteins stained with autoantibodies in total we first defined the "background patterns" of frequently immunoreactive extracellular proteins in healthy and diseased people. An assignment of these proteins, among them many nominally intracellular proteins, to the subset of exosomal proteins within the secretomes revealed a large overlap. On this basis we defined and consequently confirmed novel biomarker candidates such as the extreme C-terminus of the extracellular matrix protein agrin within the set of cancer-enriched immunorectivities.

**Conclusions:**

Our findings suggest, first, that autoantibody responses may be due, in large part, to cross-presentation of antigens to the immune system via exosomes, membrane vesicles released by tumor cells and constituting a significant fraction of the secretome. In addition, this immunosecretomics approach has revealed novel biomarker candidates, some of them secretome-specific, and thus serves as a promising complementary tool to the frequently reported immunoproteomic studies for biomarker discovery.

## Background

Serological screening methods have been used extensively to identify autoantigens in autoimmune diseases and in cancer. Various experimental approaches have been developed that exploit the humoral immune response in cancer patients to indicate tumor associated antigens. The experimental methods used and the results obtained are summarized in a number of recent excellent reviews [1-6]. For more than two decades, numerous groups made use of the SEREX (serological profiling of tumor antigens) technology where recombinant expression libraries are screened with cancer patient sera. In order to represent more closely the natural sources of immune responses in cancer patients, including protein modifications, the proteome of tumors has, in recent years, been used as the antigen source for autoantibody profiling. This approach called SERPA (serological proteome analysis), AMIDA (autoantibody mediated identification of antigens) or Proteomex involves performing 2D-Western blots on tumor lysates using human cancer sera as the source of antibody. It is also undertaken with array platforms in order to increase throughput.

From all of these studies it has become apparent that autoantibody profiles are highly diversified. This has recently been illustrated by work from Li et al., who showed by 2D Western blotting that the serum from each healthy person comprises an individual pattern of numerous autoantibodies detecting a highly distinct set of antigens plus some common frequent targets [7]. Thus, the task of identifying tumor-specific autoantibodies of cancer patients above the background of "constitutive" autoantibody repertoires of unaffected individuals is most challenging. Apart from their inherent diagnostic value, serum antibodies can be regarded as indicators of novel tumor biomarker proteins, namely their cognate antigen proteins.

Here, we introduce a novel immunoscreening approach tentatively termed "immunosecretomics" which particularly aims at the discovery of biomarker proteins released by tumor cells (in vitro and) in vivo. Divergent from and complementary to currently performed proteome based immunoscreening studies we make use of the extracellular proteome of tumor cells. The extracellular proteome, tentatively termed the secretome and defined as the entirety of proteins in conditioned media (cleared from cellular debris and apoptotic bodies) is composed of proteins specifically released through classical secretion and unspecifically released through cell death. Moreover, prominent constituents of the secretome are delivered through ectodomain shedding of transmembrane proteins [8]. Lastly, membrane vesicles termed exosomes represent an important component of the extracellular proteome. Exosomes are derived from an endosomal compartment and are released via fusion of multivesicular bodies with the plasma membrane [9-13]. The secretion of exosomes was first described in immune cells and was later found to occur in other cell types including tumor cells [14] and intestinal epithelial cells [15].

For immunoscreening, the extracellular proteome of colorectal cancer cells was resolved on 2D gels, immobilized on PVDF membranes and used for serological screening with sera from colorectal cancer patients as compared to healthy controls. The rationale behind this approach was twofold: firstly, the release of proteins from tumors is one of the presumptive mechanisms initiating an immune response in cancer patients. In particular, cross-presentation of tumor-derived antigens to the immune system via engulfment of tumor-derived exosomes by dendritic cells has been demonstrated [16]. Secondly, the secretome in general is regarded as an enriched source for biomarker discovery [8,17-23]. The mechanisms listed above that underlie protein release *in vitro *are functional *in vivo*, as well, and contribute to the set of proteins most likely reaching the circulation.

In this study, we have performed the immunosecretomics approach described above using individual sera from 21 colorectal cancer patients and from 24 control individuals. Autoantibody patterns were assigned to a secretome master map and two groups of proteins were further analysed: First, to shed some light on common features of autoantibody profiles those proteins that most frequently elicit an immune response in general were identified by mass spectrometry. As many of these were nominally intracellular proteins, we experimentally addressed the hypothesis, that frequently detected antigens were released through exosomes. Subsequently, we focussed on the candidate tumor associated antigens, namely those proteins displaying more frequent immune reactivities in the cancer sera group. These tumor-associated autoantigen candidates were assessed by duplex Western blotting with the combination of an antibody directed against the protein of interest and serum samples positive for this candidate autoantigen as a high stringency criterion for antigen confirmation. Ultimately, we defined two novel biomarker candidates, namely Glod4, a poorly characterized glyoxalase-domain containing protein and a C-terminal fragment of agrin, a prominent large heparansulfate proteoglycan resident in basement membranes.

## Methods

### Collection of serum samples from patients with colorectal cancer and healthy donors

The 45 patients included in our study were recruited from the German university hospital, Knappschaftskrankenhaus Bochum. The personal data of patients were passed out in an encrypted form by the clinical personal data management software. All patients and healthy donors had given written informed consent for use of the samples in research http://www.ruhr-uni-bochum.de/ethik/download/Deklaration_Helsinki_2008_engl..pdf, as approved by the ethics board of the medical faculty of the university. Patient and healthy donor characteristics including histopathology data, autoimmune diseases, allergies and previous malignancies were recorded in an Access database, a short compilation is given in table 1. Serum samples were obtained from patients admitted to surgery for colorectal cancer and prior to other therapy. The control group was age-matched and in order to exclude undetected colorectal cancer or adenoma was recruited from persons who had undergone colonoscopy with negative results. Venous blood samples were drawn from the cubital vein, using the Vacutainer system of BD diagnostics (BD Vacutainer^® ^SST™ Advance 2 ml tubes), kept at room temperature for 30 minutes, then centrifuged (10 min at 3000 rpm), separated and frozen at -80°C in aliquots to prevent freeze thaw cycles.

**Table 1:** Patients and numbers of autoantigens

category	UICC stage	Localisation of tumor	allergy rheumatism diabetes	gender f/m	age	number of autoantigens
**CRC**	I	Sigma-Ca		f	50	33
	II	Rectum-Ca		m	83	19
	II	Coecum-Ca		m	65	15
	II	Sigma-Ca		m	85	20
		
	**4**			**1/3**	**71 ± 17**	**22 ± 8**

	III	Rectum-Ca		m	75	28
	III	Coecum-Ca*		f	63	23
	III	Colon Ca		m	81	29
	III	Rectum-Ca		m	76	31
	III	Colon-Ca*	allergy	f	58	29
	III	Colon-Ca*	diabetes type II	m	78	28
	III	Rectum-Ca		m	70	40
	III	Rectum-Ca		m	45	29
	III	Colon-Ca		m	65	54

	**9**			**2/7**	**68 ± 11**	**32 ± 9**

	IV	Coecum-Ca		f	84	30
	IV	Rectum-Ca		m	68	27
	IV	Rectum-Ca*		m	82	28
	IV	Sigma-Ca		f	48	21
	IV	Coecum-Ca	allergy	f	51	23
	IV	Coecum-Ca	allergy	f	50	24
	IV	Coecum-Ca	allergy	f	83	39
	nd	Rectum-Ca	rheuma	m	87	26

	**8**			**5/3**	**69 ± 17**	**27 ± 6**

**all CRC**	**21**			**8/13**	**69 ± 14**	**28 ± 8**

**CONTROL**		**Colonoscopy result**				
	11	Negative		**5/6**	**55 ± 18**	**34 ± 13**
	13	Diverticulosis		**7/6**	**72 ± 7**	**35 ± 15**
			4× rheuma		**65 ± 16**	**33 ± 14**
**CONTROL**	24		10× allergy	**12/12**	**20 - 83**	**7 - 60**
			4× diabetes		**71 ± 6**	**26 ± 13**

Nd not defined; * prior malignant disease

### Cell culture and preparation of the subproteomes "secretome" and "exosome"

The human colorectal carcinoma cell lines SW948, SW620, SW480, HT29, CaCo2 were chosen for secretome production. The cell lines SW620 and SW480 were obtained from the American Type Culture Collection (Rockville, MD, USA), the cell lines HT29, SW948 and CaCo2 were kindly provided by M. Strauss/Berlin.

All cells used to produce conditioned media were grown in DMEM supplemented with 10% fetal calf serum, 2 mM glutamine, 100 U/mL penicillin and 100 mg/mL streptomycin until they reached a confluency of approximately 60-70%. Cells were then washed three times with DMEM and incubated in serum-free medium supplemented with hydrocortisone at 1 ng/mL and ITS additives (Sigma, St. Louis, MO, USA) consisting of 5 mg/mL insulin, 5 mg/mL transferrin and 5 ng/mL sodium selenite for two days. This protocol did not measurably influence the rate of cell death as determined by trypane blue exclusion. The conditioned media of serum-free cell cultures were cooled down on ice, centrifuged (200 g, 10 min) and passed through 0.2 μm pore filters to remove cellular debris, protected from proteolytic digestion by adding an inhibitor cocktail (7 nM pepstatin, 85 μg/ml PMSF and inhibitor cocktail complete™ Roche, Mannheim, Germany), and concentrated by ultrafiltration (Centriplus YM-3, Millipore). The protein concentration of secretomes was determined using a standard Bradford protein assay (BioRad, Hercules, CA, USA).

The enrichment of exosomes was performed as described by van Niel et al. [24] with modifications. In brief, secretome samples derived from the five colorectal carcinoma cell lines used here were subjected to ultrafiltration with a cut off at 100 kDa and subsequent ultracentrifugation at 120,000 × g for 1 h at 4°C using a T890 titanium fixed angle rotor (Sorvall, Langenselbold, Germany). The pellet was resuspended in PBS. Enrichment of exosomes was confirmed by Western blot analysis of secretome and exosome samples for exosomal marker proteins such as syntenin, alix, EpCAM, and Lamp3 [25].

### Two-dimensional gel electrophoresis

For 2-D-gel electrophoresis the concentrated proteins were desalted using Micro Bio-Spin 6 chromatography columns (Biorad), dried in a Speed Vac and resuspended at a final concentration of 10 μg/μl in IEF sample buffer (30 mM Tris-HCl, 2 M thiourea, 7 M urea, 4% CHAPS; pH 8.5). Dithiothreitol (1.08 g/ml, Bio-Rad) and ampholine 2-4 (GE Healthcare; Freiburg, Germany) were added to the protein samples to a final concentration of 75 mM and 2% (v/v), respectively. The protein samples were separated on 2D-gels in 11 × 14 cm dimension. Isoelectric focusing of proteins was carried out by running IEF tube gels (11 cm × 0.9 mm) with free ampholytes in a self made IEF chamber on a voltage gradient for 15,45 h according to Klose et al. [26]. The tube gels were ejected and equilibrated in 125 mM Tris buffer (with 40% w/v glycerol, 3% w/v SDS, 65 mM DTT, pH6,8) for 10 min. The SDS-PAGE (second dimension) was performed on 15.2% T, 1.2% C polyacrylamid gels. The IEF tube gels were placed onto gels 11 cm × 14 cm × 0.8 mm and fixed using overlay agarose (Biorad).

### 2D-Western blotting

Protein samples (180 μg) were separated on 2-D gels (11 × 14 cm) as described above. The secretome proteins were transferred to a PVDF membrane in a semi-dry blotting procedure. After blotting the gels were stained with silver according to Heukeshoven [27] to check for the quality of sample preparation and electrophoresis; images were scanned. Only blots with high similarity to the master gel were further proceeded. The membranes were blocked for 2 h in blocking buffer (Odyssey blocking buffer and PBS, 1:1 with Tween 0.05% v/v) and incubated with patient sera in a dilution of 1:10 in blocking buffer for one hour. An anti-human-IgG (Fc) antibody conjugated with the fluorescent dye IRDye 800 (Rockland) was used as the secondary antibody. Background "signals" resulting from this secondary antibody are shown with a Western blot performed with the secondary antibody, only (additional file 1 figure S1). The signals were detected using the Odyssey Infrared Imaging System (LI-COR Biosciences) using the same sensitivity for all blots. The threshold for a signal was set to tenfold above background. Signals were detected with values varying between 0.2 and 25 (integrated intensities).

In order to match the immunoreactive spots to the Master map, each digital Western blot together with the corresponding silver-stained gel was overlayed to the Master map using Adobe photoshop software as exemplarily shown in additional file 1 figure S2). An antigen number was assigned to each spot on the master map detected by antibodies in one or several sera. The autoantigen signature of each serum, that is, all of the signals detected on each individual Western blot, was translated into the corresponding set of antigen numbers and results were recorded in the above mentioned Access database.

### Antigen identification by MALDI-TOF/TOF-MS and nano-HPLC/ESI-MS/MS

To identify the protein spots of interest 180 μg secretome protein were separated on preparative gels with a size of 11 cm × 14 cm and 1.5 mm thick applying a voltage gradient for 14.7 h. Proteins were stained with an MS compatible silver staining procedure according to Blum [28]. The spots were manually excised and processed for mass spectrometry as previously described [8,17,29]. Tryptic peptides were analysed with MALDI-TOF/TOF-MS using an ultraflex II™ (Bruker Daltonics, Bremen, Germany) according to the manufacturer's instructions. Peptides were spotted onto an MTP AnchorChip™ 800/384 TF target (Bruker Daltonics). The target positions were manually coated with a saturated solution of α-cyano-4-hydroxycinnamic acid (HCCA) matrix. Dried samples were subsequently washed with 0.1% TFA to remove sodium and potassium adducts. The spectra were acquired in the positive mode with a target voltage of 20 kV and a pulsed ion extraction of 17.25 kV. The reflector voltage was set to 21 kV and detector voltage to 1.7 kV. Internal calibration of peptide mass fingerprint (PMF) spectra was performed using the autolysis products of trypsin (see additional file 2). PMF spectra were processed using the FlexAnalysis™ (v.2.2) software (Bruker Daltonics). The parameters used for peak-picking were based on the peak detection algorithm Snap, a signal to noise threshold of 6, a maximum number of 100 peaks with a quality factor threshold of 50. For subsequent protein identification the mass lists were sent to the ProteinScape™ database (Bruker Daltonics). Searches were started from ProteinScape™ database, using the ProFound (Knexus v. 2001.09.15) or MASCOT (v.2.2.0 and v.2.0.04) - search algorithms [30]. A ProFound score of >1.65 and a Mascot score of >64 was set as threshold for protein identification. The following search parameters were selected: fixed cysteine modification with propionamide, methionine oxidation as variable modification, one and two maximal missed cleavage sites in case of incomplete trypsin hydrolysis, mass tolerance of 50 and 100 ppm, respectively, a MW mass range from 5.0 to 250.0 kDa and pI range of 2.0 to 12.0. Searches were run using the human protein subdatabase of the NCBI (http://www.ncbi.nlm.nih.gov, June 2003-November 2006). The searches using the NCBI database were rerun to get actual gi numbers of the subdatabase (Feb - April 2008).

Peptides from protein spots with a low or no significant ProFound Score were automatically selected for MS/MS using the MALDI-TOF/TOF instrument. The obtained data were assigned with the SEQUEST™ algorithm [31]. The same search parameters as described above were used with the following exception: the peptide mass tolerance was set at 0.5 Da for monoisotopic masses and at 0.3 Da for fragment masses. A SEQUEST score of > 1.5 for a single peptide was set as threshold for protein identification. All searches were repeated using the same parameters except that the taxonomy was extended to *mammalia*, in order to identify putative contaminating bovine proteins originating from fetal calf serum in the culture media. All identified protein spots were depicted in the additional file 2. The mass lists and spectra of identified antigens described in table 2 and 3 were shown in additional file 3.

**Table 2:** Identified antigens with most frequent reactivities (PMF spectra and mass lists are given in additional files 2 and 3)

AG	Protein name	Gene symbol	PMF Profound	Accession	Seq Cov %	peptides matched (un-)	Seq pI	Seq_MW kDa	pI gel	MW gel kDa	immune reactions % (no)		Exo- somal
											CRC	control	
**12**	Triosephosphate isomerase 1 [Homo sapiens]	TPI1	2.4	gi|17389815	78.7	16	7.4	26.6	7.5	25.7	100 (21)	100 (24)	yes
**18**	Transferrin	TF	2.4	gi|15021381	35.8	26	6.7	77	6.4-7.1	75.8	100 (21)	100 (24)	yes
**14**	Enolase	ENO1	2.3	gi|4503571	33.4	13	7.4	47.2	7-7.6	55.0	100 (21)	96 (23)	yes
**14**	PA2G4 protein	PA2G4	2.1	gi|33879698	55.2	12	7.8	41.7	7-7.6	55.0	100 (21)	96 (23)	yes
**17**	Aldolase A	ALDOA	2.4	gi|4930291	53.4	16	8.3	44	8.5	45.0	76 (16)	75 (18)	no
**2**	succinate-ubiquinone oxidoreductase Ip subunit precursor	SDHB	1.7_Sequest_	gi|180917	6.1	1	9.1	30	7.8	16.5	81 (17)	75 (18)	no
**2**	UDHUP2 cystatin SN precursor [validated]-human	CST1	1.9	gi|2144579	50.4	8	7	16.4	7.8	16.5	81 (17)	75 (18)	no
**10**	peroxiredoxin 1; thioredoxin-dependent peroxide reductase 2; proliferation-associated gene A; natural killer-enhancingfactor A [Homo sapiens]	PRDX1	2.3	gi|32455266	59.3	12	8.3	22.1	8.1	22.9	57 (12)	71 (17)	no
**10**	lipocalin 2 (oncogene 24p3) [Homo sapiens]	LCN2	2.4	gi|49457137	65.2	14	9.6	22.5	8.1	22.9	57 (12)	71 (17)	no
**3**	A Chain A, Human Cyclophilin A Complexed With 2-Thr Cyclosporin	CYPA	2.3	gi|1431788	55.2	15	8.7	17.9	8.4	17.8	57 (12)	71 (17)	no
**65a**	stress-induced-phosphoprotein 1 (Hsp70/Hsp90)	STIP1	2.3	gi|5469884	20.8	11	6.4	62.6	6.3-7	66.0	57 (12)	71 (17)	yes
**13**	phosphoglycerate kinase 1/migration-inducing gene 10	PGK1	2.4	gi|41350401	61.4	25	8.3	44.7	8.2	45.0	62 (13)	67 (16)	yes
**13**	phosphoglycerate kinase 1 [homo sapiens]	PGK1	2.4	gi|129902	63.4	14	8.3	44.7	8.2	45.0	62 (13)	67 (16)	yes
**19**	Albumin (bovin)	ALB	2.4	gi|30794280	32.9	25	5.8	69.3	5.8-6	67.0	76 (16)	67 (16)	no
**44a**	enolase 1 variant	ENO1	2.3	gi|62897945	49.8	16	7.7	47.1	6.1	40.5	38 (8)	63 (15)	yes
**44a**	enolase 1 variant [Homo sapiens]	ENO1	2.3	gi|62896593	46.3	17	7.7	47.2	6.1	40.5	38 (8)	63 (15)	yes
**30**	Phosphoglycerate mutase 1 (brain) [Homo sapiens]	PGAM1	2.3	gi|56081766	72.8	15	6.7	28.8	7.1	28.3	62 (13)	54 (13)	yes
**30**	Phosphoglycerate mutase 1 (brain) [Homo sapiens]	PGAM1	2.2	gi|38566176	48.4	9	6.8	28.8	7.1	28.3	62 (13)	54 (13)	yes
**11**	Proteasome subunit MB1	PSMB5	2.3	gi|30582393	63	13	7.5	26.9	8.2	24.0	57 (12)	50 (12)	yes
**26**	peroxiredoxin 2 isoform b [Homo sapiens]	PRDX2	2.4	gi|33188452	54.4	9	6.2	16	5.6	22.9	43 (9)	46 (11)	yes
**26**	J Chain J, Thioredoxin Peroxidase B From Red Blood Cells	PRDX1	2.1	gi|9955016	57.4	14	5.6	21.6	5.6	22.9	43 (9)	46 (11)	yes
**4**	Cofilin 1 (non-muscle) [Homo sapiens]	CFL1	2.3	gi|15126676	46.4	7	8.3	18.5	8.7	15.9	(52 (11)	46 (11)	yes*

* Not detected in overlay exosome sample and secretome master (see Figure 4), but detected in other experiments and literature

**Table 3:** Antigens more frequently reactive with CRC patient sera

AG	Protein name	Gene symbol	PMF Profound	Accession	Seq Cov %	peptides matched (un-)	pI Seq	Seq_MW kDa	pI gel	MW gel kDa	Immune reactions % (no)		Diff fisher p < 0.05	Exo somal
											CRC	control		
**39**	Not identified by MALDI, *1 but nano HPLC-ESI	PSMA1,	217^m^	IPI00871889.1	18.6	4	6.6	30.2	6.8	27.8				
		KLK6	117^m^	IPI00023845.1	12.7	3	7.9	26.9	6.8	27.8	48 (10)	17 (4)	*0,0509*	yes
**31**	Phosphoglycerate mutase 1 (brain)	PGAM1	2.4	gi|56081766	73.2	16 (23)	6.7	28.8	7.5	28.1	66 (14)	33 (8)	*0,0256*	yes
			2.4											
**74a**	Lectin, mannose-binding 2	LMAN2	2.3	gi|16878112	38.5	11 (6)	6.3	40.2						
	**UV excision repair protein**	RAD23B *1	124^m^	IPI00642549.2	7.4	3	5.2	42.3	5.7	32.4	29 (6)	0	*0,0067*	no
	RAD23 homolog B*1													
**41**	syntenin isoform 3	SDCBP	71.5	gi|55749515	53.2	10	7.9	32.3	7.6	32.6	38 (8)	21 (5)	No 0,3234	yes
**41**	syntenin isoform 1	SDCBP	71.4	gi|55749490	53	10	6.8	32.4	7.6	32.6	38 (8)	21 (5)	No 0,3234	yes
**41**	syntenin [homo sapiens]	SDCBP	71.4	gi|2795863	53	10	6.8	32.4	7.6	32.6	38 (8)	21 (5)	No 0,3234	yes
**23**	Agrin precursor	AGRN	0.6	IPI00374563.2	0.6	7	6	214.7	5.2	23.6	19 (4)	4 (1)	No 0,169	no
**23**	AGRN_HUMAN Agrin	AGRN	2.2^s^	gi|61218463	0.6	1	6	214.7	5.2	23.6	19 (4)	4 (1)	No 0,169	no
**23**	AGRN protein [Homo sapiens] carboxyterm of agrin precursor	AGRN	65*	gi|21706410	59.1	7 (37)	5.5	19.5	5.2	23.6	19 (4)	4 (1)	No 0,169	no
**49**	serine (or cysteine) proteinase inhibitor, clade B (ovalbumin),	SERPINB1	2.2	gi|62898301	30.3	12 (22)	5.9	42.7	6.1	46.2	24 (5)	4 (1)	No 0,0831	yes
**49**	aminoacylase 1 [Homo sapiens]	ACY1	2.4	gi|12653545	34.8	11 (3)	5.8	45.9	6.1	46.2	24 (5)	4 (1)	No 0,0831	yes
**62a**	Lectin, mannose-binding 2	LMAN2	2.3	gi|16878112	32.6	9 (33)	6.3	40.2						
	Glyoxalase domain-containing protein 4*1	Glod4 *1	244^m^	IPI00007102.3	12.1	5	7.7	55	5.4	33.1	42 (9)	8 (2)	*0,0132*	no
		(C17orf25)		IPI00792035.1	26.4	5	10	25.8						
**24/24a**	similar to huntingtin interacting protein 2;	HIP2	2	gi|4885417	32.5	7 (21)	5.2	22.5	5.0	23.6	24 (5)	8 (2)	No	yes
**24/24a**	B Chain B, Ubiquitin-Conjugating Enzyme E2-25 kDa	HIP2	2.0	gi|60594412	37.6	7 (9)	5.2	22.5	5.1	24.2	24 (5)	8 (2)	No	yes
			2.4											
**24/24a**	Crystal Structure Of Pi Class Glutathione Transferase	GSTP1	1.8	gi|11514458	66	12 (19)	5.3	32.1	5.1	24.2	24 (5)	8 (2)	No	yes
			2.4	gi|39654104										
**70**	TALDO1 protein	TALDO1	2.4	gi|48257056	32.1	10 (15)	6.4	37.4	6.6	39.2	v			
			2.4											
**72**	The Ran-Gppnhp-Ranbd1 Complex	RANBD1	1.9	gi|5107684	41.7	9 (22)	9	23.2	7.8	26.4	19 (4)	4 (1)	No	yes
			1.9											
**25b**	Glutathione Transferase Mut	GSTP1	2.4	gi|39654104	66.1	12 (19)	5.3	23.1	5.5	24.6	19 (4)	0	No	yes
**69b**	ALDOC protein	ALDOC	2.4	gi|41351364	37.6	11 (12)	6.5	42.5	7.2	44.0	14 (3)	0	No	yes
**81b**	glyoxalase I	GLO1	2.4	gi|16198506	70.7	16 (17)	5.1	20.7	4.6	24.8	9,5 (2)	0	No	yes*

* Not detected in overlay exosome sample and secretome master (see Figure 4), but detected in other experiments and reported as exosomal in the literature ^m^Mascot score, ^S ^Sequest score; n.d. not described; *1 further identifications by nano-ESI-HPLC see supplementary Table 4

Antigens not identified by MALDI-MS(/MS) or not confirmed by duplex Western blots with specific antibodies were reanalyzed by nano-HPLC/ESI-MS/MS on a high capacity ion trap instrument (HCT plus, Bruker Daltonics). To this end, tryptic peptides were generated and extracted twice from the gel with 50% ACN/2.5% formic acid [50:50 (v/v)]. Online reversed-phase capillary HPLC separations were performed on a Dionex LC Packings HPLC system (Dionex LC Packings, Idstein, Germany) as described by Schäfer et al. [32]. The mass spectrometer was operated in the sensitive mode with the following parameters: capillary voltage 1400 V; end plate offset, 500 V; dry gas, 10.0 l/min; dry temperature, 160°C; aimed ion charge control 150000; maximal fill-time 500 ms. The nano-ESI source (Bruker Daltonics, Germany) was equipped with distal coated SilicaTips (FS360-20-10-D; New Objective). MS spectra were the sum of seven individual scans ranging from *m/z *300 to *m/z *1400 with a scanning speed of 8,100 (*m/z*)/s. Data-dependent software (HCT plus, Esquire Controle, Bruker Daltonics, Germany) was employed to select the two most intense, multiple-charged peptide ions detected within the MS spectra to subsequently conduct MS/MS fragmentation analysis. Low energy collision-induced dissociation (CID) was performed on isolated peptide ions by applying a fragmentation amplitude of 0.6 V. Generally, MS/MS spectra were the sum of four scans ranging from *m/z *100 to *m/z *2200 at a scan rate of 26,000 (*m/z*)/s. Exclusion limits were automatically placed on previously selected mass-to-charge ratios for 1.2 min. The ion trap instrument was externally calibrated with commercially available standard compounds.

Peaklists of MS/MS spectra were generated using the software Data-Analysis 3.3 with default parameters. For peptide and protein identification, peaklists were correlated with the human International Protein Index (Human IPI V3.54) http://www.ebi.ac.uk database containing 75426 protein entries using MASCOT (release version 2.2.0) [33]. All searches were performed with tryptic specificity allowing one missed cleavage. Oxidation of methionine was considered as variable modification. Mass spectra were searched with a mass tolerance of 1.2 Da for precursor ions and 0.4 Da for fragment ions and MS/MS spectra were accepted with a minimum MASCOT score of 20. Proteins were assembled on the basis of at least two peptides and a false discovery rate (FDR) of 0% using the ProteinExtractor Tool (version 1.0) in ProteinScape (version 1.3, Bruker Daltonics). The FDR was calculated as described [34] and is the quotient of the number of all proteins identified in a shuffled database and the sum of all protein identifications in both the human IPI database and its shuffled version. Details of the antigens (data and spectra) identified by nano-HPLC/ESI-MS/MS were depicted in additional file 4.

### Confirmation of autoantigen identification by duplex Western blotting

Western blotting with individual human serum samples was performed as described above using a secondary antibody conjugated with the fluorescent dye IRDye 800. Subsequently, the blot was reprobed with one of the specific antibodies recognizing syntenin (Synaptic Systems 133002, 1:1000), PGAM1 (Abcam, ab 2220, 1:1000), Aldolase C (D14; Santa Cruz sc-12066; 1:1000), Vip36 (V-20; Santa Cruz, sc-32441; 1:1000) or Agrin (K-17; Santa Cruz, sc-6166; 1:250), respectively. These primary antibodies were detected with species-specific secondary antibodies (goat, rabbit or mouse) each conjugated with the fluorescent dye Alexa Fluor 680 (Rockland). Signals derived from the human serum and from the specific antibodies were scanned at the appropriate wavelengths with the Odyssey Infrared Imaging System (LI-COR Biosciences) and can be depicted separately or combined.

### Statistical analysis

The significance of differential immunreactivities of patient versus control sera with a particular antigen was tested with Fisher's exact test with a p-value of 0.05. The data analyses were performed using GraphPad Prism version 4.00 for Windows (GraphPad Software, San Diego California USA, http://www.graphpad.com).

## Results and discussion

### Autoantibody profiling on 2D Western blots of secretome proteins with sera from colorectal cancer patients as compared to healthy controls

For autoantibody profiling serum samples were obtained from 21 patients, diagnosed with colorectal cancer and prior to therapy. As a reference to the identification of disease-specific autoantibodies, we used serum samples from 24 age-matched control individuals, all of whom were negative for any other known malignancy and precancerosis. Patient and control individual characteristics are listed in Table 1.

To establish autoantibody profiling for colorectal cancer we initially performed 1D Western blotting using cell lysates and secretomes in parallel. Whereas the patterns obtained with the secretome as the antigen source appeared less complex as compared to the pattern on cell lysates we also observed secretome-specific reactivities (data not shown). We have reported previously, that the "secretome" here defined as the entirety of proteins released into the conditioned media represents a distinct subproteome displaying some overlap with the corresponding cell lysate but also harbouring numerous secretome-specific proteins (additional file 1 figure S3). Moreover, we observed that the secretomes derived from individual cell lines significantly differed from each other (data not shown). In order to obtain a representative colorectal cancer secretome, we decided to use five human colorectal cancer cell lines for secretome production and to pool the secretomes in equal amounts. A comparison of the antigen pattern derived from test sera on this pooled secretome sample resolved on a two-dimensional gel as compared to tumor cell lysates confirms that our approach is complementary to the "standard" immunoscreening methods in current use (data not shown). The secretomes were subsequently used for autoantibody profiling by 2D Western blotting with patient and control sera.

**Figure 1:**
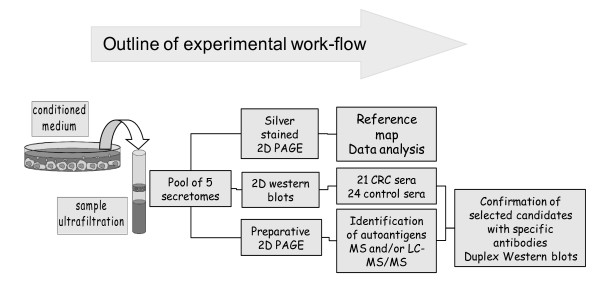
**The immunosecretomics workflow**. Secretomes are prepared from five human colorectal carcinoma cell lines. A 2-dimensional secretome master map is established. Corresponding 2-dimensional Western blots are prepared using individual sera from CRC patients and healthy controls to indicate autoantigens. Signals derived from autoantibodies in the sera are matched to the master map, corresponding proteins are excised from preparative gels and identified by mass spectrometry and database searches. Ultimately, the autoantigen candidates are confirmed by duplex Western blotting with human sera and with specific antibodies directed against the protein of interest.

**Figure 2:**
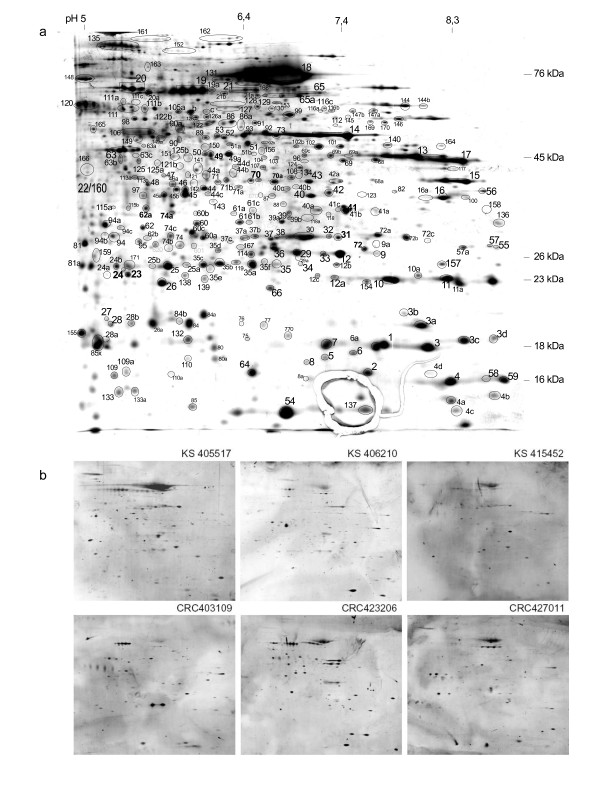
**The secretome master map and exemplary 2-D Western blots**. Secretome proteins are separated by 2-D PAGE and stained with silver to serve as a master map (a). Numbers indicate the spots detected by one or several sera on corresponding 2-D Western blots. Figure 2b provides examples of 2-dimensional Western blots with individual sera from CRC patients and healthy controls.

The experimental work-flow is depicted in Figure 1. In brief, a secretome master map was prepared by silver staining of the secretome pool resolved on a 2D gel (Figure 2a). All 21 patient and 24 control sera were investigated individually by 2D Western blotting for the presence of antibodies to secretome proteins. All of the signals from every blot were assigned to the master map and autoantigen candidates were isolated from preparative gels. Protein identification was performed by MALDI-MS and by nano-HPLC/ESI-MS/MS. Selected candidates were confirmed by Western blotting with specific antibodies.

The nature and condition of the antigen source used for immunoscreening as well as details of the experimental protocol strongly influence the results of a particular study. This study significantly varies from previously published approaches in several aspects: Firstly, we have used the Odyssey system for signal detection. Primary antibody reactivities (in the case of this study: serum autoantibodies) are detected by a secondary antibody directly coupled with a fluorescent dye and signals are obtained with a near-infrared light scanner. In contrast to the currently used chemoluminescence based systems this detection method is independent of enzymatic signal amplification and allows for semiquantitative assessment of signals. More importantly, signals can be detected whose intensities vary for three to four orders of magnitude. We believe this to be an invaluable advantage, in particular when complex patterns of autoantibody reactivities are determined on 2D Western blots. Secondly, the Odyssey system allows for signal detection in two channels and enables duplex Western blotting. Thus, confirmation of autoantigen candidates via Western blotting with a specific antibody can be performed with unprecedented exactness, as human serum reactivities and signals derived from the specific antibody can be detected on the same blot. Lastly, where unequivocal protein identification via MALDI-MS was not feasible or where candidates were disproven we here performed additional nano-HPLC/ESI-MS/MS analyses of proteins cut from 2D gels as apparent single protein spots. Results presented below illustrate that this approach in combination with duplex Western blot confirmation can prevent misinterpretation of results.

Western blotting with individual sera showed that each serum - whether patient or control - gave rise to an individual complex autoantibody profile on the colorectal cancer secretome (see Figure 2b for examples). All proteins that stained with autoantibodies - 281 in total - were assigned to a master map of the colorectal carcinoma cell secretome and the detection frequency of each antigen with patient versus control sera was plotted. The number of antigens detected by each serum sample is shown in additional file 1 figure S4 for the control and the CRC group, respectively and provided in table 1 for the individual CRC sera. The majority of the sera displayed between 20 and 40 signals. We cannot confirm previous reports by some researchers that cancer patients possess more or stronger autoantibody reactivities as compared to controls. Rather, five of the six serum samples which show more than 50 signals are from the control group (compare additional file 1 figure S4). In addition, we did not detect any dependency of antigen numbers on age or gender of the serum donors. Moreover, diseases associated with autoimmune processes like rheumatism and diabetes as well as allergies which were represented in the cancer and control groups in a few cases did not detectably impinge on the frequency of autoantigen detection.

**Figure 3:**
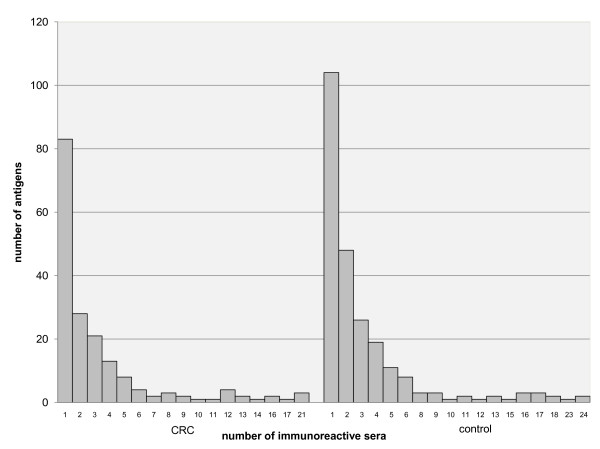
**Diversity of autoantibody signatures**. The numbers of antigens reactive with 1, 2, 3 and more sera are plotted individually for the control and the CRC group, indicating a higher diversity of autoantibodies in the group of healthy controls.

Each antibody reaction derived from each serum was assigned to a protein spot on the master gel (see methods section for details and additional file 1 figure S2 for illustration). Together, the patient sera detected 179 different protein spots, the control sera 240; bringing the total number of protein spots/autoantigens detected in this study to 281 (all reactivities for each serum sample are individually shown in additional file 5). Of these 281 autoantigens, about 30% were detected only once by one individual serum, each, and more than 50% of antigens were detected by 2-10 sera; 10% of antigens were detected by 10-20 of the 45 serum samples and 16% of proteins were detected by the corresponding autoantibodies in more than 20 sera. These results are consistent with a study by Li *et al*. who analysed autoantibody profiles of healthy Chinese people and reported that a very broad spectrum of self components could be recognized by the immune system [7]. When we plotted autoantibody frequencies separately for the cancer and control group, we observed a higher diversity of autoantibodies in the group of control sera (Figure 3).

### A large fraction of autoantigens displaying frequent immunoreactivity are exosomal proteins

We were most interested in two aspects of this autoantibody profile: firstly, which proteins most frequently elicit an autoantibody response in general and secondly, which antigens most frequently elicit autoantibodies in the disease group? Correspondingly, we aimed to identify, firstly, those antigens detected by more than 20 sera in total and secondly, those antigens, which are more frequently detected by patient sera. The corresponding protein spots were assigned to preparative gels, the spots were isolated and tryptic peptides analysed by MALDI mass spectrometry and database searches.

Antigens detected by more than 20 sera are listed in table 2; another 45 antigens identified by mass spectrometry are listed in additional file 2. Three proteins, namely transferrin, triosephosphate isomerase and enolase are detected by virtually each serum sample. The detection of transferrin by autoantibodies in every serum sample may be due to the experimental design: large amounts of transferrin (roughly one fourth of the total protein, added as a culture medium supplement) are present in the secretomes and are presented to the patient samples. Triosephosphate isomerase and enolase were also detected by 45/45 and 44/45 serum samples. Triosephosphate isomerase, enolase and many other of the frequently detected proteins are intracellular proteins. Their preponderance among proteins detected by autoantibody profiling on cellular secretomes appears surprising at first glance.

Likewise, the large overlap of proteins identified in this immunosecretomics approach as compared to other studies using cell lysates as an antigen source came as a surprise. For example, Li *et al*. who profiled 36 serum samples from healthy Chinese individuals for autoantibodies also detected enolase, phosphoglycerate mutase and triosephosphate isomerase among the most frequently immunoreactive proteins in lysates of a glioma cell line [7]. Thus, we sought for an explanation for these findings.

**Figure 4:**
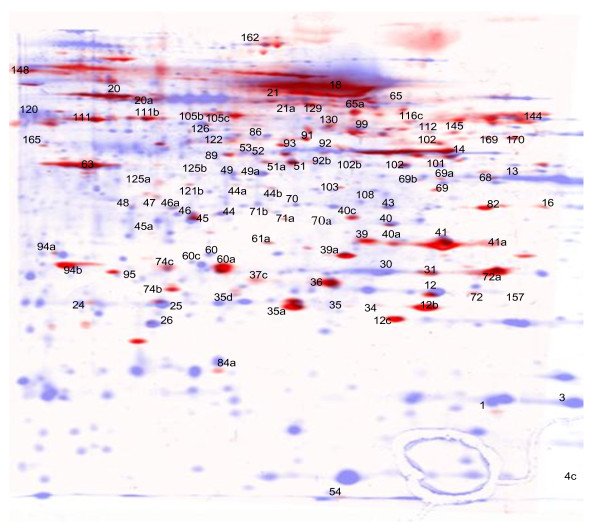
**Comparison of secretomes and exosomes**. An enriched exosome preparation was separated by 2-D PAGE and stained with silver. This exosome "master map" depicted in red was overlayed onto the secretome master map, here shown in blue using Adope Photoshop software. Frequently detected autoantigens are strongly represented in the exosome fraction (compare tables 2 and 3).

Analysing the composition of secretomes in more detail we found previously that a significant fraction of secretome proteins is contributed through the release of exosomes. We wished to experimentally analyse if the autoantigens as defined here may be of exosomal origin. To that end, we isolated the exosomal fraction of the secretome pool and performed 2D gel electrophoresis. Through an overlay of the secretome master map and the exosomal protein pattern, immunoreactive proteins were assigned to the exosome fraction (Figure 4). About one third of all antigens defined by our approach overlapped with spots in this exosomal preparation. More than 60% of these antigens belong to the frequently reactive antigen group including enolase, phosphoglycerate mutase and triosephosphate isomerase (compare Table 2). In addition, whereas aldolase, cyclophilin A and cofilin could not unequivocally be assigned to the exosomal protein fraction by this image analysis, we and others have also previously detected these three proteins in catalogues of exosomal proteins [35]. These results confirm our assumption, that the abundance of nominally intracellular proteins within the antigenic secretome proteins is due to exosomes. Moreover, these findings may underline the involvement of exosomes in the regulation of immune responses - either induction of tolerance or immune defense [36-38]. Another protein in the list of the most frequently detected antigens is the secreted protein cystatin SN; it is detected by 35 of the 45 sera. Cystatin SN belongs to a family of cystein proteinase inhibitors and is characterized as a salivary cystatin. Autoantibodies detecting cystatins, to our knowledge, have not yet been reported. Interestingly, whereas the normal intestinal epithelium does not express cystatin SN, its expression has been found in colorectal adenomas in an RNA-based profiling study [39]. The frequency of autoantibodies against cystatin SN, however, does not significantly differ between the patient and control groups.

Of interest, some of the most frequently reactive proteins listed in table 2 have previously been detected as tumor-associated antigens (compare additonal file 6 for a compilation of published antigen frequencies). For example, 47% of sera from HCC patients reacted with STIP1 [40] and 50% of sera from esophageal cancer patients detected peroxiredoxin [41], whereas the frequencies with control sera were reported with 10% and less in these studies but displayed 71% in our study. Likewhise, DeMonte et al. found 36% positivity for aldolase A with sera from colorectal cancer patients and 15% with control sera whereas 76 and 75% of CRC and control sera were positive in our study [42]. Due to the variability in experimental approaches and materials used it is not feasible to directly compare results from different SERPA-based studies. We believe, however, that the use of the secretome as a complementary antigen source and the use of the Odyssey system with its capacity to determine widely varying levels of signal intensities have a strong impact on the results.

### Identification of cancer-enriched autoantigens

On this basis we next sought to identify cancer-specific or cancer enriched autoantigens in the colorectal cancer secretome. In total, we defined protein spots which were detected with at least three more cancer than control sera or detected *exclusively *with at least two cancer sera. The corresponding proteins were isolated from preparative gels and proteins were subject to MALDI-TOF/TOF-MS. In some cases two proteins were identified from one spot; proteins from two spots - 62a and 74a - were both identified as LMAN2. Again, with the exception of spots 62a and 74a and spot 23 (identified as agrin), these proteins showing cancer-enriched immunoreactivity could be assigned to the exosomal fraction of the secretome. LMAN2 is an intracellular lectin. It is a type I membrane protein localized to vesicles that cycle between the endoplasmic reticulum (ER) and the Golgi apparatus and is involved in glycoprotein sorting and trafficking [43,44]. The theoretical and the experimental masses for LMAN2 are consistent, indicating that LMAN2 is present in the secretome as an intact, full-length protein.

Agrin, the protein identified in spot 23, is a secreted extracellular matrix protein. It is a heparan sulfate proteoglycan of more than 200 kDa in size and is a major component of basement membranes [45-47]. Here, a protein with an apparent molecular mass of about 20 kDa was identified as agrin by MALDI-MS. We hypothesized that this protein spot was due to proteolytic processing of full-length agrin. We assigned all seven peptides identified in repeated attempts of protein identification for this spot to full-length agrin and found that all of them mapped to the extreme C-terminus of approximately 200 amino acids. The theoretical pI of this fragment (5.5) is consistent with the experimental pI of 5.2. It is known that agrin is expressed by alternatively spliced mRNAs giving rise to protein products with slightly different sizes. Moreover, agrin can be cut by MMP3, which leads to the release of the C-terminal half of the protein [48]; other not yet exactly defined fragments have been reported [49]. Neither full-length agrin nor any fragments thereof have previously been identified as autoantigens before.

### Confirmation of cancer-enriched autoantigens via duplex Western blot analyses

The more our insight into the composition of cellular secretomes is increasing the more we learn about the complexity of this subproteome. Here we used medium-sized 2D gels for autoantibody profiling, the resolution of which is limited. Data compiled in tables 2 and 3 illustrate that more than one protein may reside in each protein spot isolated from preparative gels as expected. Thus, it is imperative to perform further studies for confirmation of autoantigen identification. To this aim we have used duplex Western blotting where specific antibodies to our autoantigen candidates were available.

**Figure 5:**
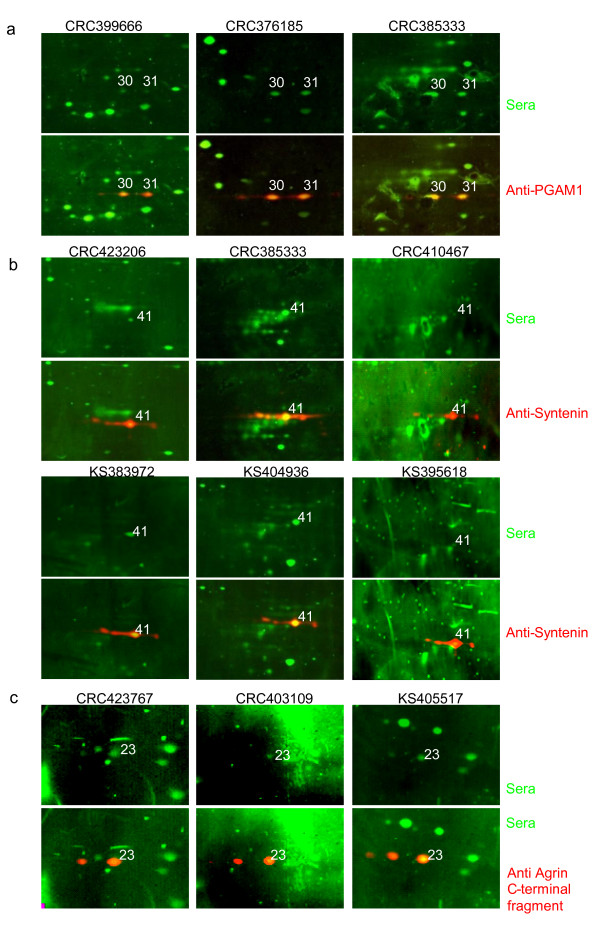
**Three examples of confirmation of antigen identification: PGAM1, syntenin and agrin by duplex Western blotting**. Spots number 30 and 31 were both identified as PGAM1 by mass spectrometry. 2-D Western blots were performed with seven individual human sera and detected with a secondary antibody conjugated with the fluorescent dye IRDye 800 as described before. The blots were then reprobed with a PGAM1-specific antiserum that was detected with a secondary antibody coupled with Alexa Fluor 680, confirming the identity of spots 30 and 31 as PGAM1 in every case. Three examples for the confirmation of PGAM1 as the antigen number 31 are shown **(a)**. Spot number 41 was reactive with 8/21 CRC and with 5/24 control sera. All positive sera were used in duplex Western blots with a syntenin-specific antiserum, confirming the correct assignment of immunoreactions for all but one (CRC) serum. Representative examples are depicted **(b)**. Spot number 23 displaying an experimental mass of 23 kDa was identified as agrin, a large glycoprotein (>200 kDa) of the extracellular matrix. Duplex Western blotting with an antiserum directed against the C-terminal fragment confirms the identity of spot 23 as an agrin fragment presumably generated by proteolytic processing **(c)**.

Spot 31 was identified as PGAM1 and displayed reactivity with 14/21 CRC sera but with 8/24 control sera, only. The adjacent spot 30 was also identified as PGAM1 and is the more frequent antigen but the 18 sera from CRC patients and controls display no difference in reactivity (13/21 and 13/24). The specific antibody detected spot 31 in seven 2D-duplex Western blots performed with seven individual sera, positive for spot 31 and spot 30 (Figure 5a). This result would usually be rated as an unequivocal confirmation of PGAM1 as a cancer enriched autoantigen. However, the specific PGAM antibody in addition to spot 31 also detects the adjacent spot 30. This finding may suggest that the reactivity of patient sera directed against PGAM1 is isoform-specific. On the other hand it cannot be excluded, that an additional protein, unrelated to PGAM1 and not yet identified may reside in spot 31 to give rise to the differential immunoreactivities of CRC versus control sera. Support for this alternative hypothesis comes from the observation, that the exosomal protein fraction displays a larger protein spot at position 31 as compared to 30 whereas the secretome, by contrast, shows a larger spot at position 30 (compare figure 4). Ongoing experiments aim to clarify this complex issue.

Spot 41, identified as syntenin, was another candidate differentially reactive with cancer versus control sera (8/21 versus 5/24 respectively), although this difference did not reach significance when a Fisher exact test was performed (table 3). Spot 41 confirmed to represent the main signal detected by a syntenin-specific antibody. All control and CRC sera positive for spot 41 were individually retested in duplex Western blots with the syntenin-specific antibody. All signals with the exception of one CRC serum were confirmed to coincide with the main syntenin spot as detected by the specific antibody (Figure 5b). In conclusion, there is no doubt that syntenin is the protein reactive with cancer and control sera in spot 41. The difference in frequency of immunoreactivity with cancer versus control sera, however, is moderate, only.

The next candidate protein on our list of cancer enriched autoantigens is the extreme C-terminal fragment of agrin (Spot 23). A purified goat polyclonal antibody directed against a C-terminal agrin peptide localised to this region is commercially available. Using this serum on duplex Western blots with three individual human sera unequivocally confirmed the identity of the agrin fragment in spot 23 (Figure 5c). In addition, a second protein spot additionally detected by the specific agrin serum at a slightly more acidic pI was also depicted by two of the three human sera.

**Figure 6:**
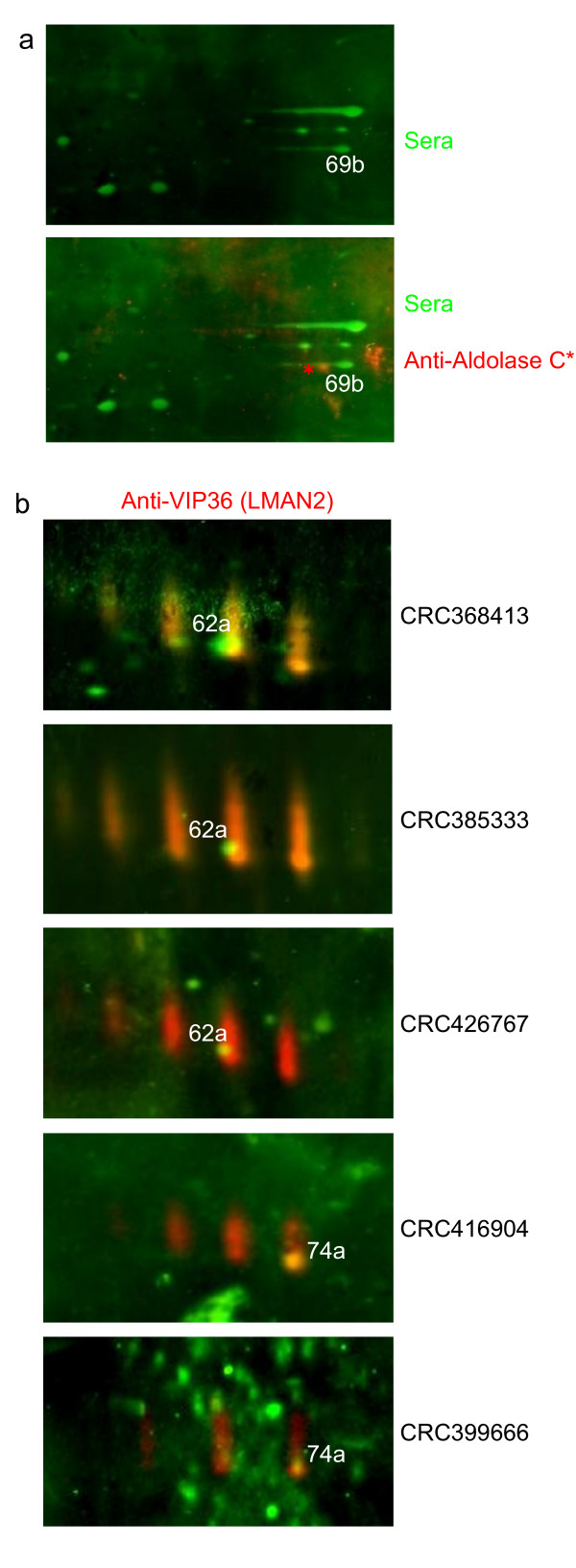
**Two examples of disproving antigens: aldolase C as the antigen in spot number 69b (a) and LMAN2 as the antigen in spots number 62a and 74a (b)**. A protein assigned to the immunoreactive spot 69b was excised from a preparative gel and identified as aldolase C. Duplex Western blotting with a human serum and an antibody directed against aldolase C does not show overlapping signals (**a**). The immunoreactive protein spots 62a and 74a were excised from a preparative gel and both identified as LMAN2 (VIP36). A VIP36-specific antibody showed a complex staining pattern consisting of a chain of four protein tracks extended in the second dimension. The spots 62a and 74a reside within or very close to one of these tracks. The staining patterns of the human sera and the VIP36-specific antibody, however, do not correspond to each other (**b**).

Spot 69b, exclusively detected with three CRC sera, was identified as aldolase C. This spot resides in a gel region with numerous spots in close proximity to each other. The aldolase C specific antibody stained a protein spot at the same molecular weight, but with a slightly different pI (Figure 6a). Further attempts for correct identification of spot 69b by mass spectrometry have been undertaken and have provided the same result: aldolase C. This finding suggests that the antigen residing in spot 69b may be another isoform of aldolase C, which is not detected by the commercial antibody.

Two spots, spots 62a and 74a, reactive with 9 versus 2 and 6 versus 0 (CRC versus control respectively) sera were identified as LMAN2 (lectin, mannose-binding 2), synonymous with VIP36. A VIP36 specific antibody had a staining pattern characteristic of a glycoprotein: a chain of four protein tracks extended in the second dimension was stained (Figure 6b). Both spots identified as VIP36 reside within these protein tracks confirming the exact assignment of the serum derived signals to the master secretome and to the preparative gel. However, the serum derived signals are clear-cut spots rather than tracks of proteins. One might speculate, that serum antibodies may detect unglycosylated LMAN2 proteins, only. However, upon duplex Western blotting it became apparent that spot 62a is also slightly shifted towards the left margin of the respective protein track (Figure 6b). In conclusion, although the assignment of both spots was highly reliable, we do not believe that the serum derived signals correspond to the LMAN2 protein.

As both spots displayed significant differences in reactivities with patient versus control sera, we further attempted to conclusively identify the autoantigen(s) corresponding to spots 62a and 74a. To that end, increased amounts of secretome proteins were resolved on another preparative gel and stained with Krypton (detectable at near-infrared wavelengths). The protein tracks characteristic for Vip36 were used for orientation, spots 62a and 74a were excised again and subject to nano-HPLC/ESI-MS/MS (see additional file 4). In both spots, LMAN2/VIP36 was again identified as expected (4 and 8 peptides, respectively). Moreover, several peptides corresponding to keratins were detected in these spots. In Spot 62a, ten peptides were identified corresponding to a protein called Glod4. This spot was detected by 9/21 patient sera, and by 2/24 control sera. Glod4 is a glyoxalase-domain containing protein also known as HC71, CGI-150 or C17orf25 and belongs to the glyoxalase I family [50]. Glod4 does not carry features of a secreted protein and we can not assign it to the exosomal fraction, either. However, evidence for its extracellular occurrence has been provided by a study of Molina et al. [51], and by Chen et al. [52] who performed proteomic analyses of human hemodialysis fluid and of pancreatic juice, respectively, in order to identify biomarker candidates. Moreover, Glod4 is also included in the reference list provided by Li et al. which comprises > 50 proteins identified as autoantigens in healthy Chinese individuals [7]. Expression patterns of Glod4, molecular functions and its role in carcinogenesis have not yet been addressed.

In spot 74a, 3 peptides were identified corresponding to Rad23b, a nucleotide excision repair protein. This scaffold protein has a central function in DNA repair and proteasomal degradation [53]. Genetic variations of nuclear excision repair proteins including Rad23b have been reported to confer an increased risk for bladder cancer [54] and are involved in the resistance of cancer cells to chemotherapeutic drugs [55]. The applicability of antigen Rad23b as a cancer biomarker will be assessed in future investigations.

## Conclusions

Immunoscreening approaches aimed at the identification of tumor-associated autoantigens have been published in large numbers. Yet, the molecular mechanisms that contribute to the profile of autoantibodies in an individual are poorly understood. Hitherto, a major conclusion from immunoscreening in healthy and diseased patients is that the immune response is a peculiarity of each individual and is highly diversified. Cancer-specific autoantibodies directed against each specific protein, on the other hand, are depicted at low frequencies, with few exceptions. Consequently, the use of autoantibodies as a diagnostic tool will need multiplexing, to reach sufficient sensitivity and specificity.

Immunoscreening may be a valuable tool for the identification of novel protein biomarkers (the corresponding autoantigens), in particular for serum-based biomarkers. Tumors are thought to release many proteins into the blood. Diagnosing cancer through serum-based analyses is therefore an attractive concept. The direct identification of cancer-specific proteins in blood samples, however, is a very compelling task, due to the complexity and to the large dynamic range of the plasma proteome. We have previously suggested the use of the extracellular proteome of cultured tumor cells as an enriched source for blood-based biomarkers. Here, we employ this specific subproteome for immunoscreening, using the humoral immune response of patients as an amplification system to indicate promising biomarker candidates.

Whereas this work to the best of our knowledge is the first to use the extracellular proteome as an antigen source for immunoscreening we find a large overlap between proteins identified in this study with those reported in previous work on tumor (cell) lysates by others (see additional file 6 for details). The appearance of nominally intracellular proteins in the extracellular proteome appears surprising at first glance. We could, however, show that most of the nominally intracellular autoantigens can be released from tumor cells as exosomal components. Exosomes are microvesicles derived from late endosomal compartments and implicated in many forms of intercellular communication [11,12,56]. As shown by Wolfers *et al*. tumor-derived exosomes can be engulfed by dendritic cells and initiate cross-presentation of tumor-specific antigens [16]. Exosomes constitute a significant part of the cellular secretome; so, high efficiency detection of exosomal proteins by serum antibodies is consistent with current knowledge. This finding also contributes to our understanding of the molecular mechanisms editing the autoantibody profile of an individual.

The immunosecretomics approach has led to the identification of known and novel biomarker candidates. Ubiquitously expressed proteins like PGAM1 and syntenin may not represent promising markers: the release of such proteins by many cell types in the body including hematopoetic cells would presumably confound sensitive detection of a tumor-specific release. This argument may also hold for Glo1 and Glod4. Interestingly, however, the overexpression of Glo1 in human colon tumors has previously been reported [57]. Although the frequency of autoantigens directed against Glo1 was low (two CRC sera, 9.5%), only, an analysis of its differential release from tumor versus normal cells presumably via exosomes should be addressed. Along the same lines Glod4 may deserve further attention as a biomarker candidate. Expression patterns of Glod4, molecular functions and its role in carcinogenesis have not yet been addressed.

A promising novel biomarker candidate identified in this study is the extreme C-terminal fragment of agrin. Agrin is a large multidomain heparan sulfate proteoglycan localized to basement membranes and expressed in several tissues [45-47]. Well described functions of agrin are the formation of neuromuscular junctions and acetylcholine receptor clustering in the central nervous system. Little is known yet about agrin's role in carcinogenesis. Interestingly, upregulation of agrin in primary liver cancers has recently been described [58]. Of note, whereas strong expression of agrin was observed in basement membranes of well and moderately differentiated cholangiocarcinomas, agrin staining was fragmented, decreased or even absent in poorly differentiated carcinomas, presumably reflecting the disintegration of the basement membrane upon local invasion processes. It is tempting to speculate, that proteolytic processing of agrin is involved in such progression-associated disappearance of agrin from basement membranes. Conversely, agrin processing products like the C-terminal fragment identified in this study as a tumor-associated antigen may be released into the circulation. This hypothesis as well as the suitability of the C-terminal agrin fragment as a biomarker will be assessed in ongoing studies.

## Abbreviations

AG: antigen; CRC: colorectal carcinoma; Ca: carcinoma; 2D: two dimensional; DIGE: 2D fluorescence difference gel electrophoresis (GE healthcare system); ESI: electrospray ionisation; KS: control sera; MW: molecular weight; MALDI: matrix-assisted laser-desorption ionisation; PMF: protein mass finger print; Seq cov: sequence coverage; SERPA: serological proteome analysis.

## Competing interests

The authors declare that they have no competing interests.

## Authors' contributions

SK-S made substantial contributions to design of the study and to drafting the manuscript and performing the gel and blot matching work, CE-M performed the Western blotting experiments and was involved in data interpretation; SK, AR-S have performed patient selection and serum collection; HD, BW, KS have performed mass spectrometry; HEM supervised mass spectrometry, WS contributed to the design of the study, IS-W is the PI, designed the study and drafted the manuscript. All authors read and approved the final manuscript.

## Pre-publication history

The pre-publication history for this paper can be accessed here:

http://www.biomedcentral.com/1471-2407/10/70/prepub

## Supplementary Material

Additional file 1 **Details of experimental procedures and results**. **Figure S1**. Background signals due to secondary antibody. 2D Western blot of secretome proteins was performed using the secondary antibody directly. **Figure S2**. Alignment of individual Western blot signals to the proteins on the Master map. The figure exemplarily illustrates the alignment of Western blot signals to the master gel by overlaying the digital pictures of the master gel (depicted in blue), the silver stain of an individual gel after blotting (in green) and the corresponding Western blot signals (in red). The patterns can be manually aligned by moving the pictures towards each other in the overlay to correct for regional differences in the gel runs. **Figure S3 **Comparison of patterns from tumor cell lysates versus secretome. A secretome sample (green) and a lysate sample (red) of a colorectal cancer cell line were resolved in the same 2D-PAGE using the DIGE technology (for details see protocols of GE healthcare and reference 8. Proteins which appeared in both samples are shown in yellow, whereas protein spots in red or green are unique to the respective sample. **Figure S4 **Total number of immune reactions per serum in the cancer and the control group. The number of antigens identified with each individual serum sample is depicted. Click here for file

Additional file 2 **Antigens identified by MALDI**. Table of calibrants and table of all identified antigens by MALDI including antigens given in table 2 and 3. Click here for file

Additional file 3 **Details of identification by MALDI**. PMF spectra and mass lists of antigens given in Table 2 and 3. Click here for file

Additional file 4 **Antigens identified by nano-HPLC/ESI-MS/MS**. Summary of data analysis and table of identified antigens. Click here for file

Additional file 5 **Compilation of individual autoantibody profiles**. Numbers of immune reactions for all individual sera. Click here for file

Additional file 6 **Autoantigens reported in the literature**. Examples of autoantigens and their frequencies reported in the literature. Click here for file
