# Supplementary material for: Immunoscreening of the extracellular proteome of colorectal cancer cells
Source: BMC Cancer. 2010 Feb 25;10:70. doi: 10.1186/1471-2407-10-70 (PMC2837015; doi:10.1186/1471-2407-10-70)

Additional file 1 figure S1

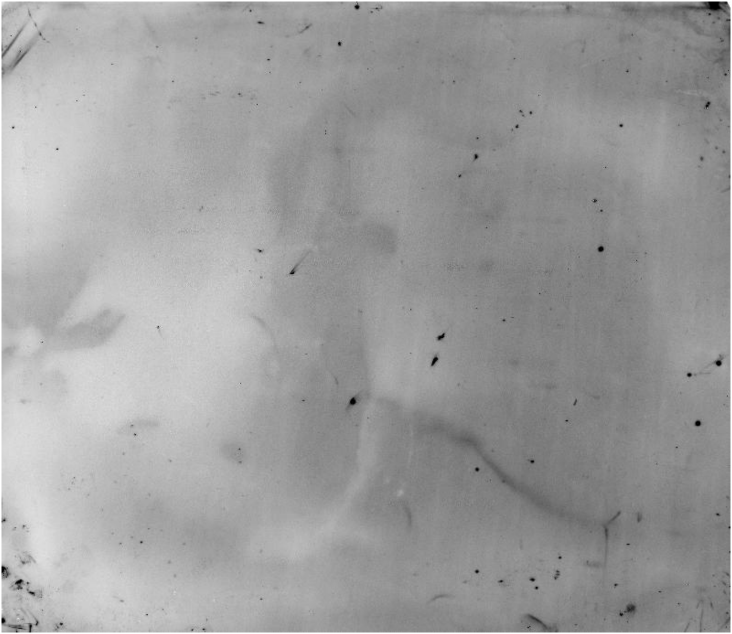

Additional file 1 figure S2

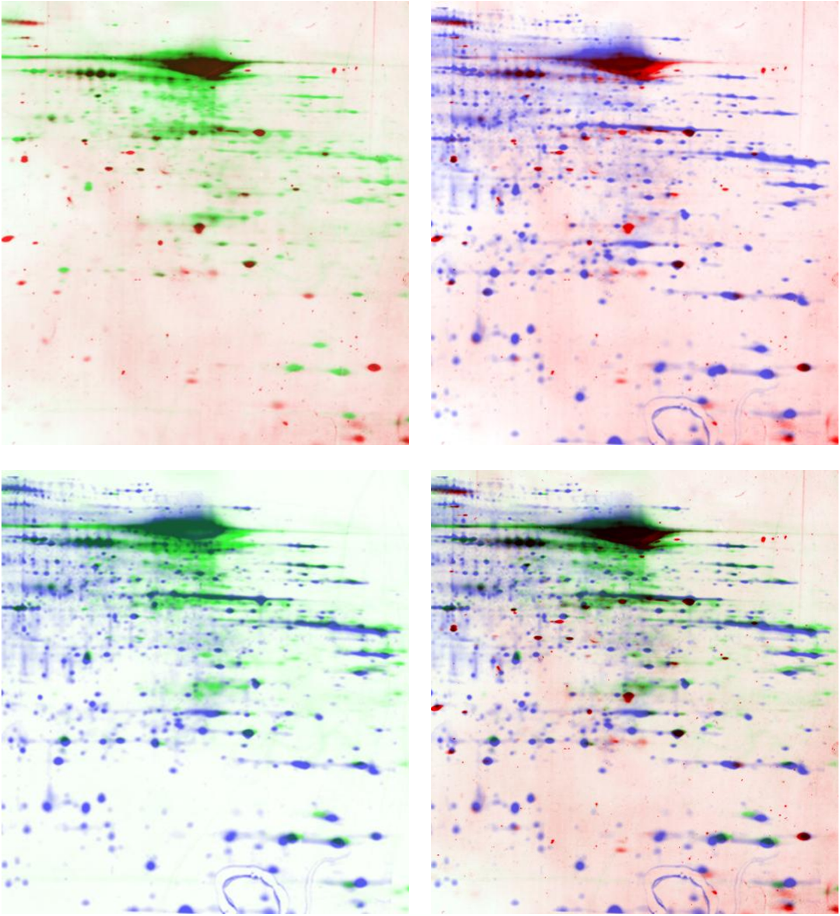

Additional file 1 figure S3

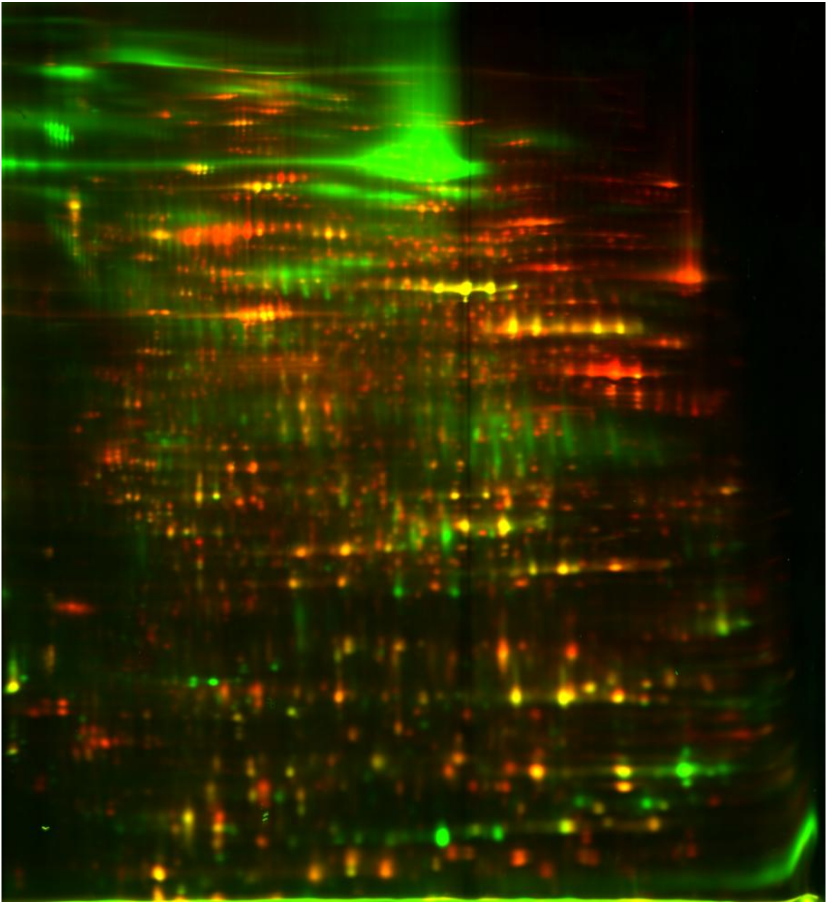

Additional file 1 figure S4

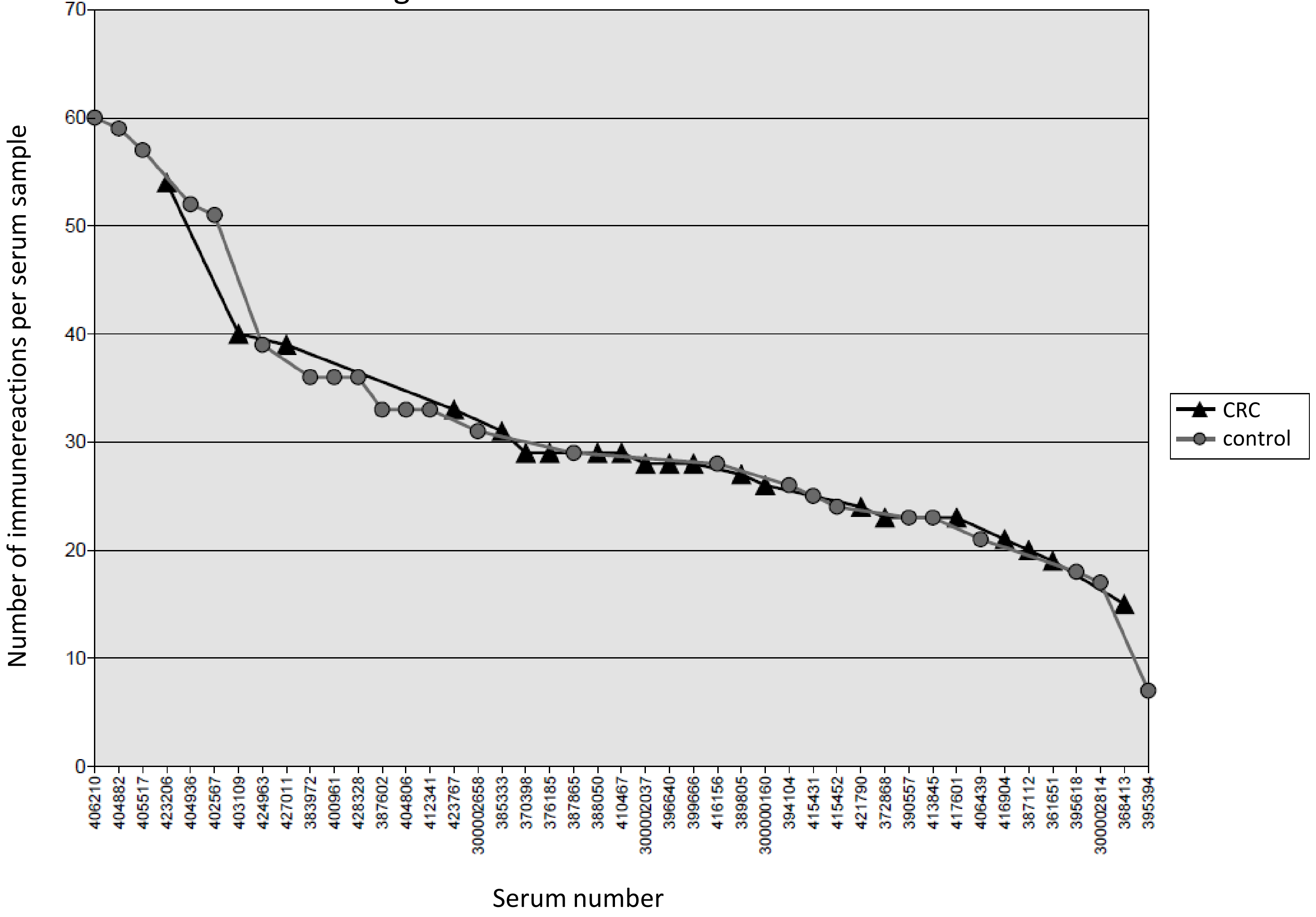

Supplement: Additional file 1 — Details of experimental procedures and results. Figure S1. Background signals due to secondary antibody. 2D Western blot of secretome proteins was performed using the secondary antibody directly. Figure S2. Alignment of individual Western blot signals to the proteins on the Master map. The figure exemplarily illustrates the alignment of Western blot signals to the master gel by overlaying the digital pictures of the master gel (depicted in blue), the silver stain of an individual gel after blotting (in green) and the corresponding Western blot signals (in red). The patterns can be manually aligned by moving the pictures towards each other in the overlay to correct for regional differences in the gel runs. Figure S3 Comparison of patterns from tumor cell lysates versus secretome. A secretome sample (green) and a lysate sample (red) of a colorectal cancer cell line were resolved in the same 2D-PAGE using the DIGE technology (for details see protocols of GE healthcare and reference 8. Proteins which appeared in both samples are shown in yellow, whereas protein spots in red or green are unique to the respective sample. Figure S4 Total number of immune reactions per serum in the cancer and the control group. The number of antigens identified with each individual serum sample is depicted. [file 1471-2407-10-70-S1.PDF]
