# Supplementary material for: Immunoscreening of the extracellular proteome of colorectal cancer cells
Source: BMC Cancer. 2010 Feb 25;10:70. doi: 10.1186/1471-2407-10-70 (PMC2837015; doi:10.1186/1471-2407-10-70)
Supplement: Additional file 4 — Antigens identified by nano-HPLC/ESI-MS/MS. Summary of data analysis and table of identified antigens. [file 1471-2407-10-70-S4.PDF]

Edit | Browse | Queries | Tools | Configuration

Su_9 Colon Pool 22- 02-08 17 Analysis.mgf Hanna_HCT(old)(modified)[Jul 03, 2009 12:27]

**MS/MS (PFF) method name**	Hanna_HCT(old)(modified)
**Search date**	Jul 03, 2009 12:27:00
**Search Engines**	Mascot (2.2.0)
**Top Proteins to report**	15
**Number of single MS/MS (PFF) spectra combined**	440
**Number of single MS/MS (PFF) with peptide results**	253
**Result Handling**	Results prepared for ProteinExtractor

Search parameters

**Fixed modifications**<br>Propionamide (C)	**Variable modifications**<br>Oxidation (M)
**Cleavage enzyme**	Trypsin (*KR)
**Max missed cleavages**	1
**Mass spectrometer**	Bruker Esquire-series (esquireHCT)

**Peptide mass tol.**	mono	1.2 Da
**Fragment mass tol.**	mono	0.4 Da

Database parameters

**Database**	ipi.HUMAN_decoy
**Taxonomy**	Homo sapiens
**MW range**	5.0 - 250.0 kDa (Sonar, Sequest)<br>250.0 kDa (Mascot)
**pI range**	2.0 - 12.0
**Protein name filter**	
**Sequence tag**	

ProteinExtractor Method

**Method Name**	070824_Micropartikel (old)
**Search engines**	Mascot
**Peptide score threshold**	20
**Weighting factors**	1
**Minimum 2 peptides with score >**	20
**Maximum number of listed proteins**	20
**Maximum MW [kDa]**	300
**Generate non-redundant protein list**	yes
**Include previously accepted homologous** [illegible]	yes

fdr = 0%

	**IPI.Human_decoy: v3.54**
**Datenbank**	**human (75426 proteins)**

Rank	Accession	Protein	MW (Seq.) [kDa]	pI (Seq.)	Seq.Cov.	Peptide Count	PE Score (Mascot)
Spot 9 = **AG 74a (6 CRC / 0 control)**							
1	IPI00009865.2	Gene_Symbol=KRT10 Keratin, type I cytoskeletal 10	59,5	5	19,9	9	533,5
2	IPI00220327.3	Gene_Symbol=KRT1 Keratin, type II cytoskeletal 1	66	8,8	11,5	7	466,9
3	IPI00009950.1	Gene_Symbol=LMAN2 Vesicular integral-membrane protein VIP36	40,2	6,5	27,2	8	450,2
4	IPI00642549.2	Gene_Symbol=RAD23B cDNA FLJ56531, highly similar to UV excision repair protein RAD23 homolog B	42,3	5,2	7,4	3	124,5
5	IPI00022463.1	Gene_Symbol=TF Serotransferrin	77	7	3,3	2	95,9
Spot 10 = **AG 62a (9 CRC / 2 Control)**							
1	IPI00792035.1	Gene_Symbol=GLOD4 Isoform 3 of Glyoxalase domain-containing protein 4	25,8	7,7	26,4	5	244,7
2	IPI00007102.3	Gene_Symbol=GLOD4 Uncharacterized protein C17orf25	55	10	12,1	5	244,7
3	IPI00009950.1	Gene_Symbol=LMAN2 Vesicular integral-membrane protein VIP36	40,2	6,5	15,7	4	165,9
4	IPI00022463.1	Gene_Symbol=TF Serotransferrin	77	7	4,3	2	129,9
Spot 11 = **AG 113a (3 CRC / 0 control)**							
1	IPI00009950.1	Gene_Symbol=LMAN2 Vesicular integral-membrane protein VIP36	40,2	6,5	15,2	4	225,4
2	IPI00387168.1	Gene_Symbol=PCSK9 Isoform 1 of Proprotein convertase subtilisin/kexin type 9	74,3	6,1	3,3	2	124,1
3	IPI00441414.1	Gene_Symbol=GANAB Isoform 3 of Neutral alpha-glucosidase AB	96,2	5,4	3,3	2	51,6
Spot 12 = **AG 48 (4 CRC / 4 control)**							
1	IPI00873484.1	Gene_Symbol=CAPZA1 Putative uncharacterized protein CAPZA1 (Fragment)	31,8	5,8	39,1	8	433,6
2	IPI00220327.3	Gene_Symbol=KRT1 Keratin, type II cytoskeletal 1	66	8,8	8,5	5	349,1
3	IPI00009865.2	Gene_Symbol=KRT10 Keratin, type I cytoskeletal 10	59,5	5	11,6	5	270
4	IPI00009950.1	Gene_Symbol=LMAN2 Vesicular integral-membrane protein VIP36	40,2	6,5	8,4	3	133,9
5	IPI00019359.3	Gene_Symbol=KRT9 Keratin, type I cytoskeletal 9	62,1	5,1	5,8	2	93,8
Spot 13 = **AG 45a (6 CRC/ 4 control)**							
1	IPI00015018.1	Gene_Symbol=PPA1 Inorganic pyrophosphatase	32,6	5,5	29,1	7	381
2	IPI00022463.1	Gene_Symbol=TF Serotransferrin	77	7	7,9	3	156,8
Spot 14 = **AG 39 (10 CRC/ 4 control)**							
1	IPI00022463.1	Gene_Symbol=TF Serotransferrin	77	7	25,8	25	1470,7
2	IPI00019359.3	Gene_Symbol=KRT9 Keratin, type I cytoskeletal 9	62,1	5,1	8,3	3	262,4
3	IPI00220327.3	Gene_Symbol=KRT1 Keratin, type II cytoskeletal 1	66	8,8	10,2	4	232,6
4	IPI00016832.1	Gene_Symbol=PSMA1 Isoform Short of Proteasome subunit alpha type-1	29,5	6,2	19	4	217,8
5	IPI00023845.1	Gene_Symbol=KLK6 Isoform 1 of Kallikrein-6	26,8	7,9	12,7	3	117,6
Spot 15 = **AG 70a (2 CRC/0 control)**							
1	IPI00009865.2	Gene_Symbol=KRT10 Keratin, type I cytoskeletal 10	59,5	5	12,5	6	460,2
2	IPI00022463.1	Gene_Symbol=TF Serotransferrin	77	7	13,5	8	355,7
3	IPI00007118.1	Gene_Symbol=SERPINE1 Plasminogen activator inhibitor 1	45	6,8	17,4	6	315,7
4	IPI00220327.3	Gene_Symbol=KRT1 Keratin, type II cytoskeletal 1	66	8,8	11,6	5	249,3
5	IPI00019359.3	Gene_Symbol=KRT9 Keratin, type I cytoskeletal 9	62,1	5,1	10,9	3	174,1
6	IPI00744692.1	Gene_Symbol=TALDO1 Transaldolase	37,5	6,4	9,8	3	174
7	IPI00016915.1	Gene_Symbol=IGFBP7 Insulin-like growth factor-binding protein 7	29,1	9,6	10,3	2	93,9
8	IPI00305380.3	Gene_Symbol=IGFBP4 Insulin-like growth factor-binding protein 4	27,9	7	10,5	2	47,3
Spot 16 = **AG 70 (4 CRC/ 0 control)**							
1	IPI00220327.3	Gene_Symbol=KRT1 Keratin, type II cytoskeletal 1	66	8,8	18,8	13	740,8
2	IPI00019359.3	Gene_Symbol=KRT9 Keratin, type I cytoskeletal 9	62,1	5,1	18,3	9	622,9
3	IPI00009865.2	Gene_Symbol=KRT10 Keratin, type I cytoskeletal 10	59,5	5	14,3	8	550
4	IPI00022463.1	Gene_Symbol=TF Serotransferrin	77	7	10,7	6	230
5	IPI00790784.2	Gene_Symbol=SERPINA1 Isoform 2 of Alpha-1-antitrypsin	40,2	5,2	7,2	2	134
