# Supplementary material for: Immunoscreening of the extracellular proteome of colorectal cancer cells
Source: BMC Cancer. 2010 Feb 25;10:70. doi: 10.1186/1471-2407-10-70 (PMC2837015; doi:10.1186/1471-2407-10-70)
Supplement: Additional file 6 — Autoantigens reported in the literature. Examples of autoantigens and their frequencies reported in the literature. [file 1471-2407-10-70-S6.PDF]

Additional file 6: Tumor antigen and frequencies of autoantibodies reported in literature

Tumor	Autoantibody frequence %		Cancer	Method	Reference
Antigen	Tumor	Control	Tissue		
GRP94	19	5	colorectal	Serpa	De Monte L J Prot Res 2008
LamC	35	10	colorectal	Serpa	De Monte L J Prot Res 2008
AldoA	36	15	colorectal	Serpa	De Monte L J Prot Res 2008
PAI-1	60	20	hepatocellular	Serpa	Looi KS J Prot Res 2008
GAPDH	33	35	hepatocellular	Serpa	Takashima M Proteomics 2006
HSP70	46	10	hepatocellular	Serpa	Takashima M Proteomics 2006
PRDX	33	0	hepatocellular	Serpa	Takashima M Proteomics 2006
PRDX	50	7	esophagial	Serpa	Fujita Y Clin Cancer Res 2006
PRDX	3	7	colorectal	Serpa	Fujita Y Clin Cancer Res 2006
Mn-SOD	40	10	hepatocellular	Serpa	Takashima M Proteomics 2006
Beta tubulin	9	5	hepatocellular	Serpa	Le Naour F Mol Cell Proteomics 2002
NDPKA	5	0	hepatocellular	Serpa	Le Naour F Mol Cell Proteomics 2002
Creatine kinase B	5	3	hepatocellular	Serpa	Le Naour F Mol Cell Proteomics 2002
Cytokeratin 8	4	1	hepatocellular	Serpa	Le Naour F Mol Cell Proteomics 2002
Cytokeratin 18	5	5	hepatocellular	Serpa	Le Naour F Mol Cell Proteomics 2002
CRT32	10	2	hepatocellular	Serpa	Le Naour F Mol Cell Proteomics 2002
CRT32	58	7	pancreatic	Serpa	Hong SH Cancer Res 2004
Vimentin	44	0	pancreatic	Serpa	Hong SH Biomark Insights 2006
HER2	15	2	prostate	Serpa	Reviewed in Lu H Proteome Res 2008
HER2	13	5	breast	ELISA	Lu H J Proteome Res 2008
p53	10	1	breast	ELISA	Lu H J Proteome Res 2008
MUC1	20	2	breast	ELISA	Lu H J Proteome Res 2008
Annexin I, II	60	0	lung	Serpa	Brichory F PNAS 2001
PGP9.5	14	2	lung	Serpa	Brichory F Cancer Res 2001
